# Neutrophil-to-lymphocyte ratio, monocyte-to-lymphocyte ratio, and platelet-to-lymphocyte ratio in different etiological causes of thyrotoxicosis

**DOI:** 10.3906/sag-1901-116

**Published:** 2019-12-16

**Authors:** Işılay TAŞKALDIRAN, Tülay OMMA, Çağatay Emir ÖNDER, Sevde Nur FIRAT, Gönül KOÇ, Mustafa Kemal KILIÇ, Şerife Mehlika KUŞKONMAZ, Cavit ÇULHA

**Affiliations:** 1 Department of Endocrinology and Metabolism, Ankara Training and Research Hospital, Ankara Turkey; 2 Department of Geriatrics, Ankara Training and Research Hospital, Ankara Turkey

**Keywords:** Thyrotoxicosis, neutrophil, lymphocyte, monocyte, platelet, ratio

## Abstract

**Background/aim:**

The most common causes of thyrotoxicosis include Graves’ disease (GD), toxic multinodular goiter (TMNG), toxic adenoma (TA), and subacute granulomatous thyroiditis (SAT). In our study, we aimed to see whether neutrophil‐to‐lymphocyte ratio (NLR), monocyte-to-lymphocyte ratio (MLR), platelet‐to‐lymphocyte ratio (PLR), and mean platelet volume (MPV) may be helpful in the differential diagnosis of these diseases.

**Materials and methods:**

We retrospectively analyzed the hospital records of the Endocrinology Clinic of our hospital between 2016 and 2019. We included data from 66 GD, 37 TA, and 35 SAT patients. We compared the data with those of 35 healthy subjects as controls.

**Results:**

NLR, MLR, and PLR were found to be higher in the SAT group when compared to other groups. The post hoc analysis of comparison of NLR, MLR, and PLR in each group showed that NLR and PLR were significantly different in the SAT group when compared to the GD, TA, and controls groups (P < 0.001, P = 0.003, and P < 0.001 for NLR respectively and P < 0.001 for PLR in all groups). MPV levels were different between groups (P = 0.007). However, the intergroup analysis (Tukey’s test) failed to show a statistically significant difference for any of the groups. In patients with SAT, PLR and NLR were significantly higher than in the GD, TA, and control groups. MLR was also higher in SAT when compared to other groups, but the difference was not statistically significant.

**Conclusion:**

High PLR and NLR may be helpful to differentiate SAT from GD and TA, the other common causes of thyrotoxicosis.

## 1. Introduction

Thyrotoxicosis describes disorders of excess thyroid hormone with or without the increased thyroid hormone synthesis. Hyperthyroidism is a pathological disorder in which excess thyroid hormone is synthesized and secreted by the thyroid gland. The most common causes of hyperthyroidism include Graves’ disease (GD), toxic multinodular goiter (TMNG), and toxic adenoma (TA) [1]. In other causes of thyrotoxicosis such as silent thyroiditis, postpartum thyroiditis, and subacute granulomatous thyroiditis (SAT), excess thyroid hormone is not related to overfunction of the gland but comes from destructed thyroid follicles [2,3].

Differential diagnosis of thyrotoxicosis is important for treatment and a follow-up plan. SAT is a self-limited inflammatory condition in which the treatment is based on antiinflammatory drugs. However, GD and TA necessitate treatment with antithyroid agents and possibly thyroid surgery [1–3]. Differential diagnosis is based on clinical features, physical examination, and biochemical evaluation. To clarify the cause, a thyroid radioactive iodine uptake (RAI) test is recommended [2–5]. RAI is high in GD and TA but low in SAT. However, this is an expensive procedure in which the patient is exposed to radioactivity and must be partially isolated from his/her household for days. 

Neutrophil‐to‐lymphocyte ratio (NLR), monocyte-to-lymphocyte ratio (MLR), and platelet‐to‐lymphocyte ratio (PLR) have been investigated recently as new inflammatory markers in many inflammatory, cardiovascular, and malignant diseases [6,7]. Mean platelet volume (MPV) is the volume of the average circulating platelets and is a marker of platelet activation that is known to be associated with inflammation [8].

In our study, we aimed to see whether NLR, MLR, PLR, and MPV may be helpful in the differential diagnosis of GD, TA, and SAT.

## 2. Materials and methods

We retrospectively analyzed the hospital records of the Endocrinology Clinic at the Ankara Training and Research Hospital between 2016 and 2019. This study includes 66 GD, 37 TA, and 35 SAT patients. None of them had been treated. The control group included 35 healthy subjects. The study was approved by the ethics committee of the Ankara Training and Research Hospital (approval date and number: 12 December 2018–63/650).

GD is confirmed by the presence of conventional symptoms of hyperthyroidism associated with a diffusely enlarged goiter, elevated levels of free triiodothyronine (FT3) and free thyroxine (FT4), decreased thyroid-stimulating hormone (TSH) level, and positive thyroid-stimulating hormone receptor antibody (TRAb), according to laboratory results. TA is confirmed in the presence of symptoms of hyperthyroidism with laboratory, ultrasound, and radioactive iodine uptake test findings. SAT diagnosis was based on typical clinical symptoms, accelerated erythrocyte sedimentation rate (ESR), and marked depression of thyroid uptake of radioactive iodine.

Pregnant patients or those who had hematologic disease, cancer, severe renal or liver disease, ongoing infection, chronic inflammatory disease, or autoimmune disease were excluded from the study.

Demographic data of the patients and laboratory parameters at the time of diagnosis were recorded.

NLR is calculated as the absolute count of neutrophils divided by the absolute count of lymphocytes, PLR is calculated as the absolute platelet count divided by the absolute lymphocyte count, and MLR is calculated as the absolute monocyte count divided by the absolute lymphocyte count. 

Blood samples were collected from both the patient and the control group after 12 h of fasting. Hemogram samples were collected in tubes with ethylenediamine tetraacetic acid (EDTA) anticoagulant and processed within 1 h after collection. All hematologic analyses were performed using the Sysmex XN 3000 instrument analyzer (Kobe, Japan). TSH, FT3, FT4, anti-TPO (thyroid peroxidase antibody), anti-Tg (thyroglobulin), and TRAb were measured by using the electrochemiluminescence method on the Roche Cobas 8000 analyzer (Rotkreuz, Switzerland). Blood samples for hormone analyses were collected in standard gel separator tubes and performed after suitable centrifugation. All samples were tested within 1 h.

### 2.1. Statistical analysis

SPSS 15 was used for statistical analysis (SPSS Inc., Chicago, IL, USA). Normality was tested using the Shapiro–Wilk test. Data are presented as mean ± standard deviation (SD) for normally distributed variables, median (minimum–maximum) for nonnormally distributed variables, and as the number of cases (%) for nominal variables. A chi-square test was performed for categorical variables to assess differences between groups. The comparisons between groups were performed by ANOVA for parametric variables and Kruskal–Wallis test for nonparametric variables. Post hoc comparisons were evaluated with Tukey’s test for parametric variables and Bonferroni correction Mann–Whitney U test for nonparametric variables. Pearson correlation analysis was used to test for correlation of normal variables considered to be associated with inflammatory parameters, whereas Spearman correlation analysis was used for variables not showing a normal distribution. Covariance analysis ANOVA was used to remove the effects of body mass index (BMI) and age on the categorical independent variables (MPV). Values of P < 0.05 were considered significant.

## 3. Results

Demographic and biochemical data of the participants are shown in the Table. Mean ages were found to be different (P < 0.001) between groups, whereas sex distribution and BMI were similar. NLR, MLR, and PLR were significantly different between groups. Other parameters are summarized in the Table. 

**Table:** Demographic and laboratory data of the participants.

	GD patients(n = 66)	TA patients (n = 37)	SAT patients (n = 35)	Control groups (n = 35)	P**
Age, years	37.53 ± 11.68	52.16 ± 12.78	41.54 ± 8.98	45.37 ± 8.06	<0.001
Sex (F/M)	48/18	26/11	24/11	26/9	0.949
BMI (kg/m²)	26.78 ± 3.10	27.59 ± 3.19	28.11 ± 3.14	27.65 ± 2.80	0.287
WBC (10.9/L)	7.12 ± 1.71	8.45 ± 2.10	9.34 ± 2.23	7.20 ± 1.48	<0.001
Neutrophils (10.9/L)	3.68 (1.67–9.14)	4.53 (2.87–9.19	6 (3.85–14)	4.2 (1.33–7.5)	<0.001
Lymphocytes (10.9/L)	2.25 (1.1–4.39)	2.51 (1.54–4.09)	2.05 (0.84–3.94)	2.4 (1.43–3.3)	0.013
Platelets (10.9/L)	265 (176–427)	286 (187–462)	379 (241–560)	266 (178–397)	<0.001
MPV	10.39 ± 1.24	10.50 ± 1.28	9.75 ± 1.38	9.76 ± 1.06	0.007
TSH (mIU/L)	0.01 (0.003–0.08)	0.01 (0.01–0.29)	0.01 (0.01–0.17)	1.92 (0.42–4.8)	<0.001
FT4 (ng/dL)	3.67 (1.46–7.77)	1.57 (0.72–3.68)	2.96 (1.49–7.77)	1.13 (0.59–1.74)	<0.001
FT3 (ng/dL)	14.4 (1.89–32.11)	5.21 (2.82–11)	6.93 (3.3–19.55)	*	
Anti-TPO (IU/mL)	165 (0.7–933)	13.1 (0.7–1087)	13.5 (0.5–60)	11 (0.2–400)	<0.001
Anti-Tg (IU/mL)	110.5 (0.9–4000)	12.05 (0.9–240)	23 (0.9–768)	*	
NLR	1.59 (0.47–5.23)	1.75(0.79–4.03)	2.84 (1.16–10.77)	1.68 (0.93–3.75)	<0.001
PLR	116.47 (55.58–215.64)	111.05 (69.44–246.75)	178.09 (73.10–604.76)	110.83 (71.48–233.58)	<0.001
MLR	0.29 (0.13–0.83)	0.22 (0.10–0.49)	0.36 (0.13–0.85)	0.20 (0.10–0.44)	<0.001

Values are presented as median (range) or mean ± SD as appropriate. WBC = White blood cells, TSH = thyroid-stimulating hormone, Anti-TPO = thyroid peroxidase antibody, Anti-Tg = antithyroglobulin, MPV = mean platelet volume, NLR = neutrophil-to-lymphocyte ratio, PLR = platelet-to-lymphocyte ratio, MLR = monocyte-to-lymphocyte ratio, FT3 = free triiodothyronine, FT4 = free thyroxine, BMI = body mass index. *Could not be calculated due to missing data. **P-value, calculated by chi-square (for categorical variables; age), ANOVA (for parametric variables), and Kruskal–Wallis (for nonparametric variables). P < 0.05 is significant.

**Figure 1:**
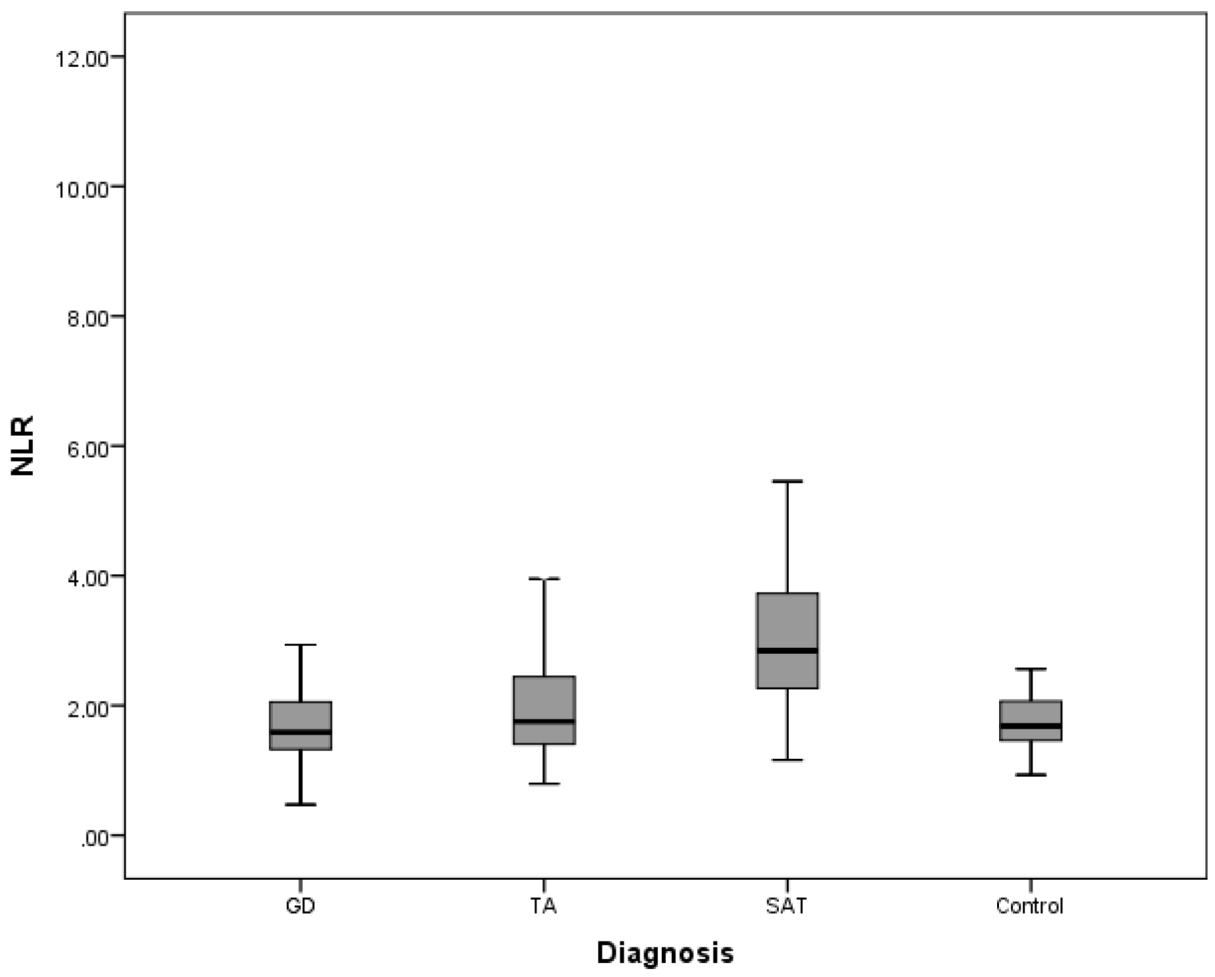

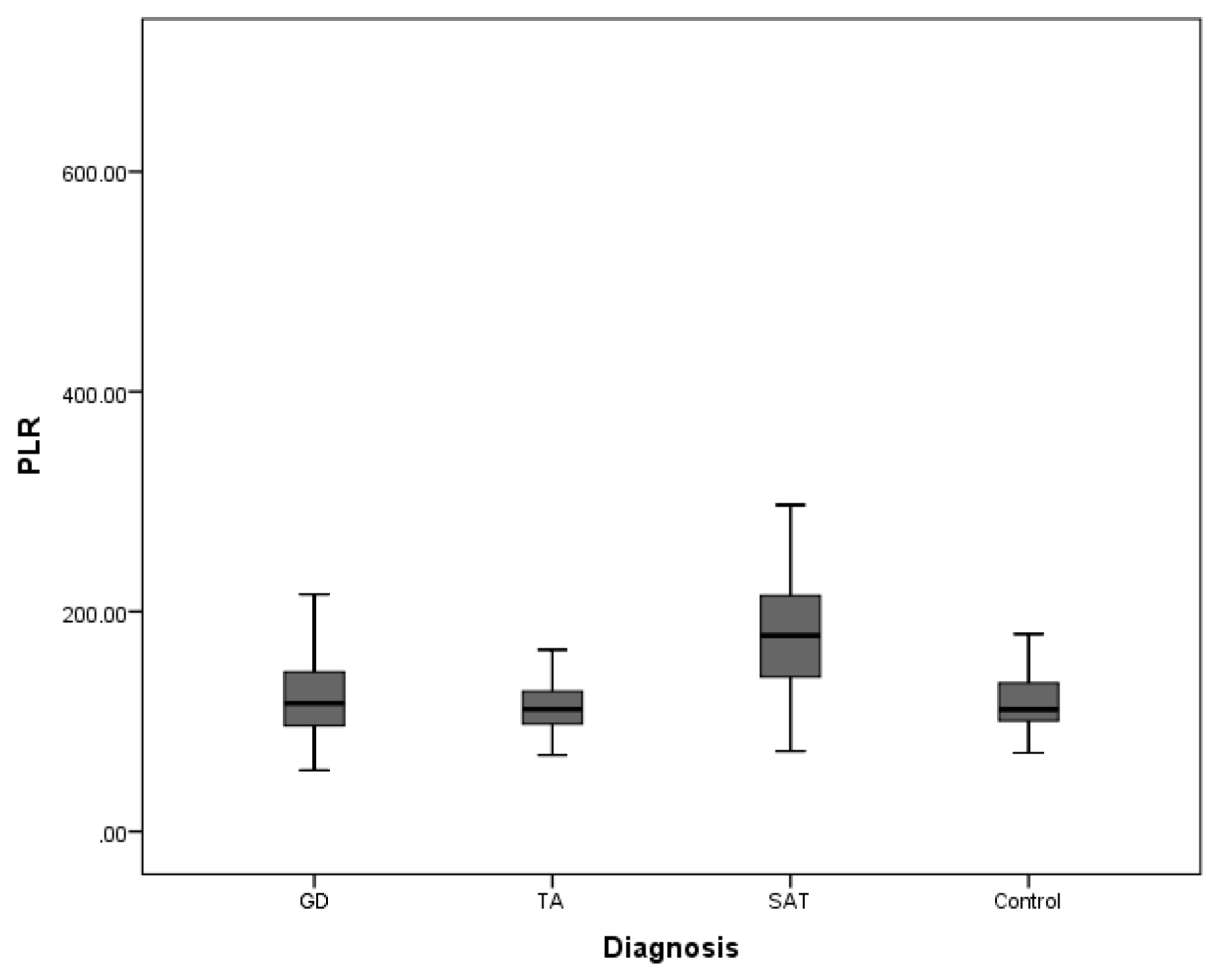

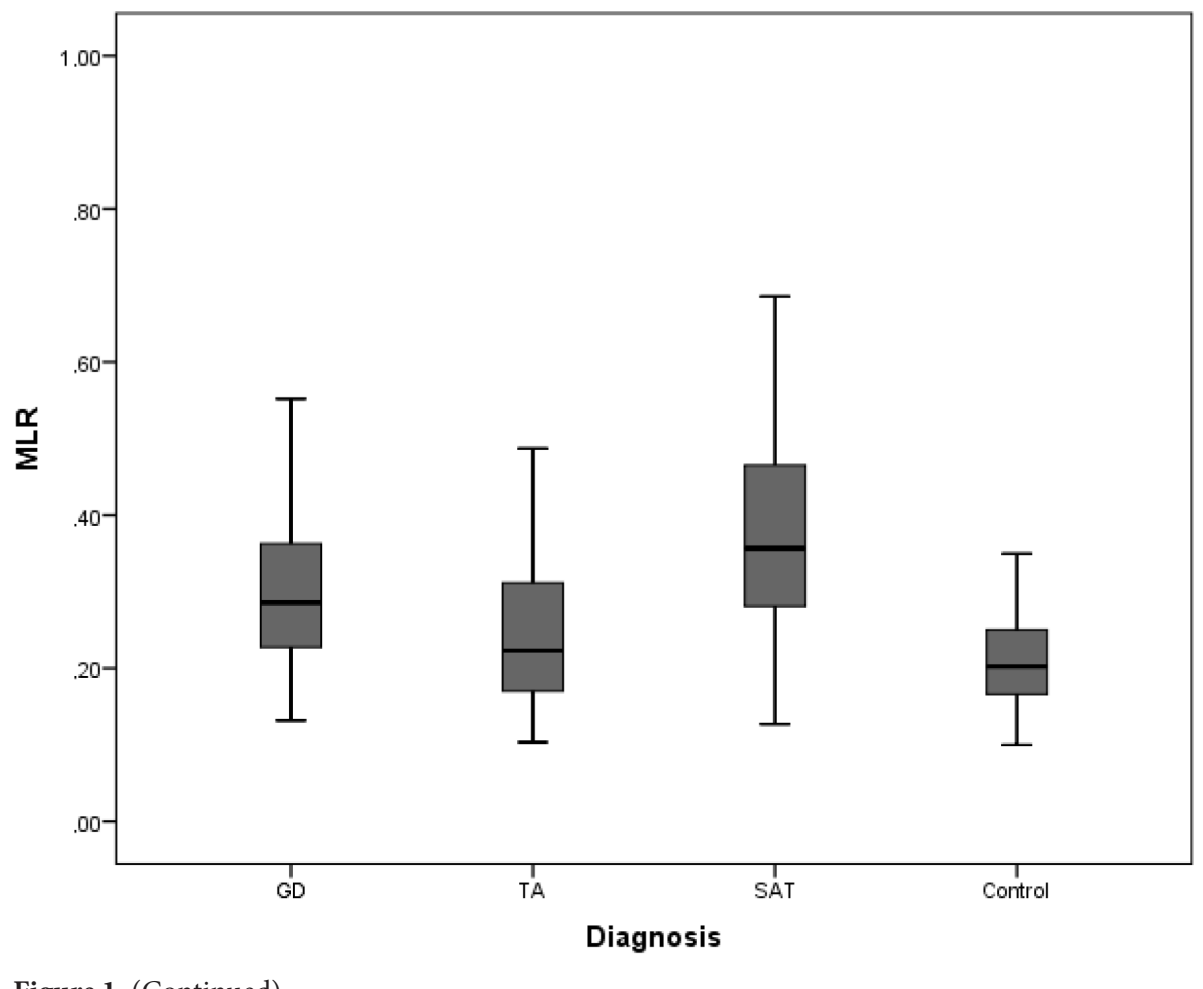
a) Comparison of NLR between groups. b) Comparison of MLR between groups. c) Comparison of PLR between groups. NLR = Neutrophil-to-lymphocyte ratio, MLR = monocyte-tolymphocyte ratio, PLR = platelet-to-lymphocyte ratio, GD = Graves’ disease, TA = toxic adenoma, SAT = subacute thyroiditis.

The inflammatory parameters tested, NLR, MLR, and PLR, are shown in Figures 1a–1c. All parameters were found to be higher in the SAT group when compared to other groups.

In post hoc analysis, NLR was significantly different in the SAT group when compared to the GD, TA, and control groups (P < 0.001, P = 0.003, and P < 0.001 respectively). PLR was significantly different in the SAT group (P < 0.001) when compared to all groups. MLR was significantly different in SAT when compared to controls (P < 0.001) and MLR was significantly different in the GD group when compared to the TA and control groups (P = 0.009, P < 0.001 respectively).

MPV levels were different between groups (P = 0.007) in post hoc analysis. However, the intergroup analysis (Tukey’s test) failed to show a statistically significant difference for any of the groups. 

There was no correlation between age, BMI, thyroid autoantibody levels, thyroid function, and the tested inflammatory markers except a positive moderate correlation between fT4 and MLR (r = 0.418). The ANCOVA test showed that MPV values were independent of age and BMI.

## 4. Discussion

NLR and PLR have been thought to be simple, inexpensive, and easily reached predictors to evaluate systemic inflammation. They have been used in several tumoral, cardiac, inflammatory, and autoimmune diseases [9]. In some studies, NLR and PLR were found to be higher in patients with rheumatoid arthritis, familial Mediterranean fever, ulcerative colitis, psoriasis, and euthyroid chronic autoimmune thyroiditis [10]. MLR was also used as a marker in malignancies and cardiac and inflammatory diseases [11]. MPV was also studied in many diseases and was claimed to be associated with inflammation [12].

GD is the most frequent cause of hyperthyroidism. GD is an autoimmune thyroid disease in which B and T lymphocytes play a major role. B lymphocytes produce antithyroid antibodies, whereas activated T lymphocytes secrete proinflammatory cytokines [13,14]. Platelets and lymphocytes are known to be reciprocally regulated in disease processes. Lymphocyte activity is modulated by platelets, soluble mediators, or direct cell-cell contact. MPV, which refers to platelet size, is related to platelet activation. Platelets actively react to the changes in their microenvironment, leading to the release of proinflammatory and prothrombotic substances. These processes cause configurational and volume changes in the platelets [15]. In our study we found that MPV was higher in GD when compared to controls, but the difference was not statistically significant. In the GD group MLR was significantly higher than controls, but NLR and PLR were similar. In a study by Dagdeviren et al., NLR was found to be similar in GD when compared to other causes of thyrotoxicosis and healthy controls [16].

TA is less common than GD, although its prevalence increases with age and in the presence of dietary iodine deficiency. Thyroid nodules become autonomous and produce thyroid hormones independent of signals from either TSH or thyrotropin-releasing hormone (TRH). Unlike GD or SAT, autoimmunity or acute inflammation does not play a role in the pathogenesis of TA [17]. In our study, MLR, PLR, NLR, and MPV levels were found to be similar to those of controls as expected.

SAT is an inflammatory disease characterized by painful goiter, thyrotoxicosis, accelerated ESR, and depressed thyroidal radioactive iodine uptake. SAT typically occurs a few weeks after a viral infection. SAT is more common in women than in men, with a ratio of 1.9–6:1, and generally seen in the third to fifth decades [18]. Microscopically, early infiltration into polymorphonuclear leukocytes is replaced by lymphocytes and macrophages, and the normal follicles may be replaced largely by an inflammatory reaction. In time, monocytes and macrophages also play a role in the pathogenesis [19]. As a result, it is clear that the neutrophils, lymphocytes, monocytes, and macrophages play an active role in the pathophysiology of SAT. In this study we showed that MLR, PLR, and NLR were all higher in patients with SAT when compared to the GD, TA, and control groups. Among these, the difference in NLR and PLR was statistically significant. This may be related to the activation of monocytes and platelets early in the disease process as well as an increase in the number of platelets [20].

There are limitations to our study. First, we conducted a retrospective study. Second, mean ages were different between groups. This is probably related to the differing age distribution of these different diseases. GD is more common in the third to fourth decades whereas toxic adenoma is seen in older patients. Unfortunately, the control group is not age-matched when all three different etiologies of thyrotoxicosis are considered. However, the tested parameters are found to be independent of age in correlation analysis, so we think that higher NLR and PLR in SAT cannot be attributed to age.

In our study, we evaluated and compared NLR, MLR, PLR, and MPV in newly diagnosed thyrotoxic patients. We think that NLR and PLR may be helpful to differentiate SAT from other causes of thyrotoxicosis before going further with more expensive and difficult diagnostic studies such as radioactive iodine uptake. Larger studies are needed to see if these new inflammatory markers may be helpful in the diagnosis of GD and other causes of thyrotoxicosis.
